# STUDY ON THE ROLE AND MECHANISM OF MICRORNA-650/WNT1 IN THE REPAIR OF ARTICULAR CARTILAGE INJURY

**DOI:** 10.1590/1413-785220243204e278218

**Published:** 2024-10-07

**Authors:** Hui Liu, Yue Wang, Shuyuan Wang, Bo Yang, Di Sun, Shuangyang Han

**Affiliations:** 1. Peking University Third Hospital, Qinhuangdao Hospital, Department of Nursing, Qinhuangdao, Hebei Province, China; 2. Peking University Third Hospital, Qinhuangdao Hospital, Department of Orthopedics, Qinhuangdao, Hebei Province, China

**Keywords:** Osteoarthritis, microRNA, Osteocondritis, Osteoartrite, microRNA, Osteocondritis

## Abstract

**Objectives:**

Osteoarthritis (OA) is a degenerative disease associated with chondrocyte injury. This study investigated the dysregulation of microRNA-650 (miR-650) in cartilage tissues of patients with OA. Its function and mechanism were also investigated in OA cell models.

**Methods:**

miR-650 levels were examined in 15 OA cartilage tissues and ten healthy cartilage tissues. SW1353 cells were used for cell function experiments and IL-1β was applied to the cells to mimic OA conditions in vitro. Cell functions such as proliferation, apoptosis, and inflammation were detected. The downstream target gene of miR-650 was identified and confirmed by bioinformatic analysis and luciferase activity assay. Rescue experiments were performed to verify the mechanism.

**Results:**

Suppressed expression of miR-650 was tested in patients with OA and cell models. Overexpression of miR-650 increased cell proliferation but suppressed apoptosis and inflammation of SW1353. As the target gene of miR-650, WNT1 overexpression counteracted the role of miR-650 in the function of SW1353.

**Conclusion:**

miR-650 can protect against articular cartilage injury in OA by targeting WNT1. ***Level of Evidence I, Experimental Study.***

## RESUMO

**Objetivos:**

A osteoartrite (OA) é uma doença degenerativa acompanhada de lesão dos condrócitos. Este estudo examinou a desregulação do microRNA-650 (miR-650) nos tecidos da cartilagem de doentes com OA. A sua função e mecanismo também foram explorados em modelos celulares de OA.

**Métodos:**

Os níveis de miR-650 foram examinados em 15 tecidos de cartilagem de OA e em 10 tecidos de cartilagem normal saudável. As células SW1353 foram utilizadas para experiências de função celular, e a IL-1β atua sobre as células para imitar as condições da OA in vitro. Foram detectadas funções celulares, incluindo a proliferação, a apoptose e a inflamação. O gene alvo a jusante do miR-650 foi reconhecido e confirmado por meio de análise bioinformática e ensaio de atividade da luciferase. Foram efetuadas experiências de recuperação para verificação do mecanismo.

**Resultados:**

Foi testada uma expressão oprimida do miR-650 tanto em doentes com OA como em modelos celulares. A sobreexpressão do miR-650 aumentou a proliferação celular, mas suprimiu a apoptose e a inflamação da SW1353. Como gene alvo do miR-650, a sobreexpressão do WNT1 contrariou o papel do miR-650 na função do SW1353.

**Conclusão:**

O miR-650 pode proteger contra a lesão da cartilagem articular na OA através da ação sobre o WNT1. ***Nível de Evidência I, Estudo de Experimental.***

## INTRODUCTION

 Osteoarthritis (OA) is a common degenerative disease that occurs primarily in older adults. ^1^ With the aging of the population, the incidence of OA has continued to increase. ^2^ Clinically, OA is characterized by arthralgia and limited mobility. The pathological features of OA include degeneration and damage to articular cartilage, subchondral sclerosis or cystic degeneration, and hyperplasia of the articular marginal bone. OA affects the joints of the knees, hips, ankles, hands, and spine, among other body regions. ^3^ More than half of people over the age of 65 will develop OA, which seriously reduces the quality of daily life. ^4^ Various factors contribute to the onset of OA, such as age, obesity, injury, etc. ^5^ The pathogenesis has not yet been elucidated. 

 As endogenous non-coding single-stranded RNAs, microRNAs (miRNAs) can mediate the expression of target genes at the post-transcriptional level. ^6^ Recent studies have indicated that miRNAs participate in the pathogenesis of OA, affecting bone metabolism, inflammation, and cartilage homeostasis. ^7^ Using miRNA microarray platform and bioinformatics, researchers have identified numerous differentially expressed miRNAs in OA samples. ^8^^,^^9^ Circulating miRNAs such as miR-206, miR-140-3p, and miR-146a serve as promising biomarkers in OA pathogenesis. ^10^^-^^12^ Recently, overexpression of miR-650 has been suggested to limit proliferation and inflammation of rheumatoid arthritis fibroblast-like synoviocytes. ^13^ Alterations in miR-650 expression levels in rheumatoid arthritis (RA) have implicated that it is an attractive molecule for OA progression. ^13^ However, few studies have highlighted the influence of miR-650 on OA. 

In this study, miR-650 levels in cartilage tissues of OA cases were examined clinically. In addition, SW1353 cells were used for cell function experiments, and IL-1β was applied to the cells to mimic OA conditions in vitro. Functionally, the effect of miR-650 on cell proliferation, apoptosis and inflammation was investigated. In terms of mechanism, the downstream target gene of miR-650 was predicted and its functions were verified.

## MATERIALS AND METHODS

### Clinical sample collection

The study design was approved by the Ethics Committee of Peking University Third Hospital Qinhuangdao Hospital. All participants signed an informed consent form.

A total of 15 patients suffering from OA were selected as the case group, and their cartilage tissues were collected during total knee arthroplasty. The case group consisted of nine males and six females with a mean age of 55.60 ± 5.93 years. Another ten participants without OA were recruited as the control group. These subjects—seven males and six females—suffered from accidental injuries and required amputation. Their normal cartilage tissues were obtained during surgery. Comparison of age and gender distribution showed no obvious discrepancy between the two groups involved, which proves that they were comparable.

### Ethical approval

This study complied with the guidelines of the Declaration of Helsinki and was approved by the Medical Ethics Committee of Peking University Third Hospital Qinhuangdao Hospital (No. 2021-05-16). All participants signed an informed consent form.

### Cell culture and modeling

 Human chondrosarcoma (SW1353) was obtained from the Cell Resource Center of Shanghai Institute of Biological Sciences (Shanghai, China). DMEM complete medium (containing 10% FBS and 1% double antibody) was used for cell culture at 37℃ and 5% CO _2_ . Samples were divided into two groups: control group and model group. Cells in the control group maintained the routine culture in DMEM, while cells in the model group were treated with IL-1β at the concentration of 10 ng/mL. Different time gradients were set, including 3 h, 6 h, 12 h, 24 h. 

### Cell transfection

To mediate the levels of miR-650 and its target gene WNT1 in SW1353, cell transfection was performed. Sequences of miR-650 mimic and its negative control (mimic-NC) were synthesized by Sangon Biotech. Sequences of WNT1 were cloned into pcDNA-3.0 vector to establish the gene overexpression plasmid, while the empty vector served as a negative control (pcDNA-3.0). After SW1353 was cultured with IL-1β for 24 hours, the above sequences were added. After incubation for 6 hours, the normal medium was changed. Cell transfection was completed after 48 hours.

### qRT-PCR

 Total RNA was extracted using TRIZOL reagent, and the RNA concentration was determined. Reverse transcription was performed using transcriptase kit (Takara Bio, Japan). SYBR Green PCRMaster Mix (Takara Bio) was used with ABI StepOnePlus real-time PCR system (Applied Biosystems, Thermo Fisher Scientific, CA) to perform qPCR. Relative values of miR-650 and WNT1 mRNA were obtained by 2 ^-△△CT^ based on CT values using U6 and GAPDH as internal references, respectively. The experiment was repeated three times. 

### CCK-8 assay

 Cell proliferation was measured using the Cell Counting Kit-8 (CCK-8) assay. The prepared cell suspension was inoculated into 96-well plates to achieve 3×10 ^3^ cells in each well. Over the course of 3 days, 10 µL CCK-8 reagent was added to each well every 24 h (DojindoMolecular Technologies, Japan). After continuing the culture in the incubator for 3 h, cell proliferation was assessed by measuring the absorbance at 450 nm. 

### Flow cytometry assay

SW1353 cells were digested with pancreatic enzymes, and single cell suspension was collected and processed. After three washes with pre-cooled PBS, 10 µL Annexin V and propyl iodide (PI) were added to the cell suspension and incubated at 4℃ in the dark. FACSCalibur flow cytometry (BD Biosciences) was used to test cell apoptosis.

### Elisa assay

Enzyme-linked immunosorbent assay (ELISA) was performed. The levels of cytokines TNF-α and IL-1β in the cell culture supernatant were tested according to the ELISA instructions. The concentration was calculated based on the OD values at 450 nm.

### Luciferase reporter assay

WNT1 wild-type or mutant (WT/MUT) sequences were transfected into logarithmic 293T cells with miR-650 mimic or mimic-NC. A total of 6 h after transfection, the fresh culture medium was replaced. Then, 48 h after transfection, luciferase activity was analyzed using double luciferase detection reagent.

### Statistical methods

 SPSS 24.0 statistical software was used to process and analyze the data. Measurement data were expressed as mean ± standard deviation (SD), and differences between groups were compared by t-test. One-way analysis of variance (one-way ANOVA) was used to compare multiple groups. Statistical significance was set at *P* < 0.05. 

## RESULTS

### Differentially expressed miR-650 in OA

**Figure 1.**
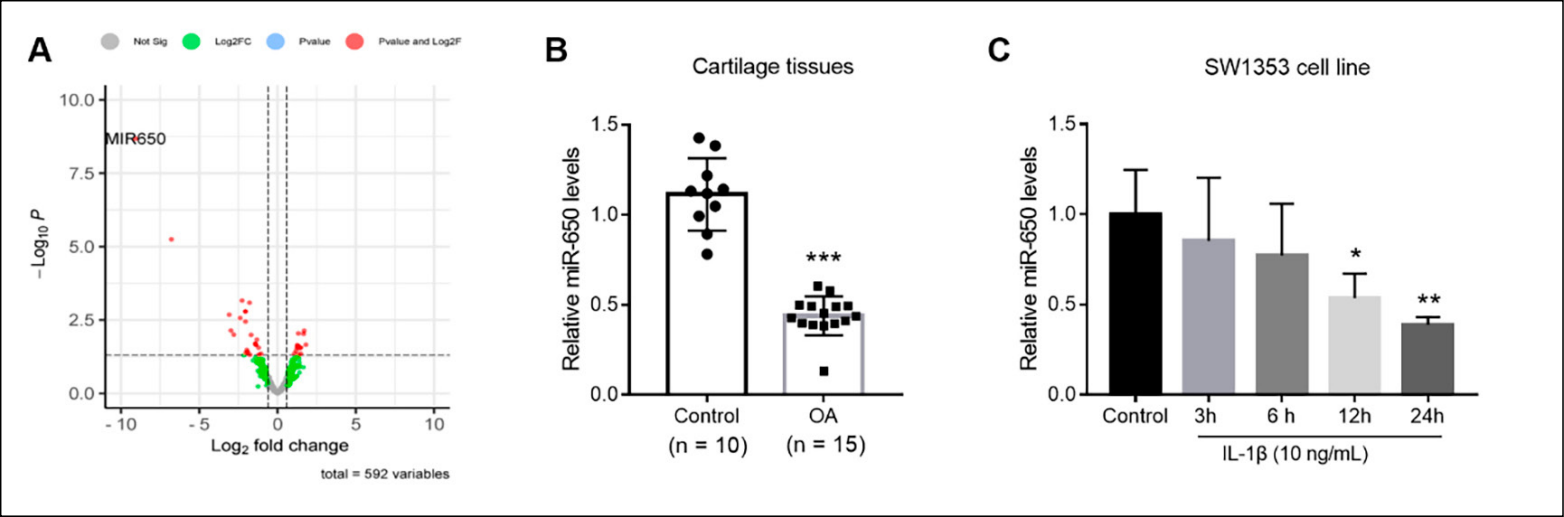
Differentially expressed miR-650 in OA. Figure A: Based on the GSE213070 dataset, differentially expressed miR-650 was identified in the inflamed synovial membrane after anterior cruciate ligament and/or meniscus injury. Figure B: Suppressed expression of miR-650 in cartilage tissues of patients with OA. Figure C: Gradual downregulation of miR-650 in SW1353 cells with increasing incubation time in IL-1β. * P < 0.05, ** P < 0.01, *** P < 0.001.

 In this study, the miRNA dataset GSE213070 was downloaded from the GE database ( https://www.ncbi.nlm.nih.gov/geo/ ). ^14^ A total of 590 differentially expressed miRNAs were detected, of which 271 were downregulated and 319 were upregulated in inflamed synovial membrane after anterior cruciate ligament and/or meniscus injuries ( Figure 1 A). MiR-650 was ultimately selected for further experiments because it had the largest fold change and the lowest *P* value. Clinically, miR-650 levels in cartilage tissues of patients with OA were analyzed and compared with the controls. As shown in Figure 1 B, a suppressed expression of miR-650 was identified in patients with OA ( *P* < 0.001). SW1353 cells were cultured with added IL-1β to mimic OA conditions in vitro. As shown in Figure 1 C, miR-650 levels gradually decreased with increasing incubation time. After 12 hours of culture, the difference reached a significant level ( *P* < 0.05). 

### Overexpression of miR-650 promoted cell proliferation and suppressed apoptosis of SW1353

**Figure 2.**
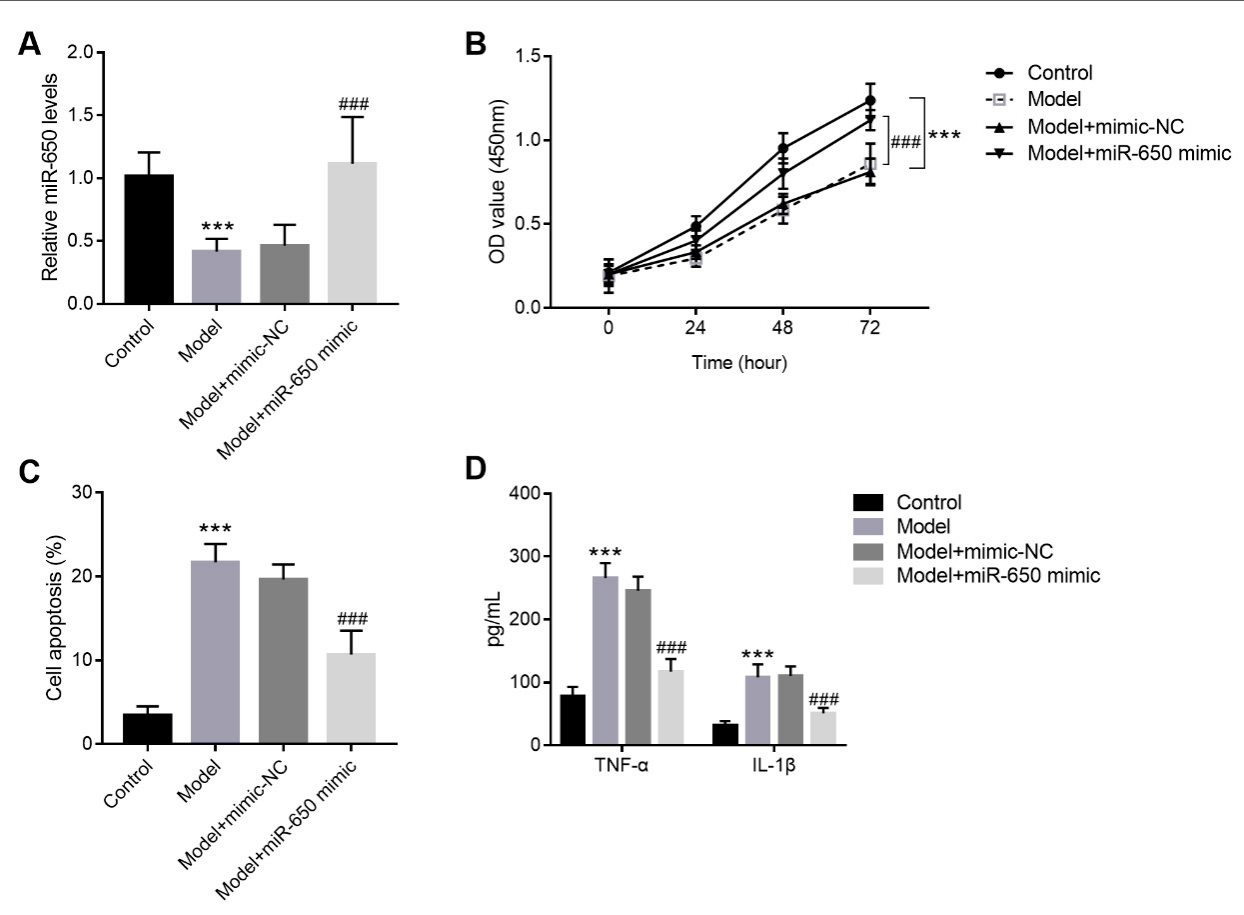
miR-650 overexpression protects against chondrocyte injury in SW1353. Figure A: The expression of miR-650 was significantly upregulated in the miR-650 mimic transfection group. Figure B: Cell proliferation of OA cell models after transfection with miR-650 mimic. Figure C: Cell apoptosis of OA cell models after transfection with miR-650 mimic. Figure D: Concentration of TNF-α and IL-1β in SW1353 after treatment with IL-1β and/or transfection with miR-650 mimic. *** P < 0.001 vs. control group; ### < 0.001 vs. model group.

 To investigate the role of miR-650 in OA, miR-650 mimic was transfected into SW1353 to mediate its levels in vitro. As shown in Figure 2 A, miR-650 levels were significantly upregulated in the miR-650 mimic transfection group ( *P* < 0.05). The CCK-8 results indicated that miR-650 overexpression enhanced the proliferation capacity of SW1353 compared with the model group ( Figure 2 B). The results of cell apoptosis were tested by flow cytometry assay, and it was seen that cell apoptosis was remarkably suppressed after miR-650 mimic transfection ( Figure 2 C). Similarly, the reduction of TNF-α and IL-1β in SW1353 was accompanied with miR-650 mimic transfection ( Figure 2 D). 

### miR-650 directly targets WNT1 binding

**Figure 3.**
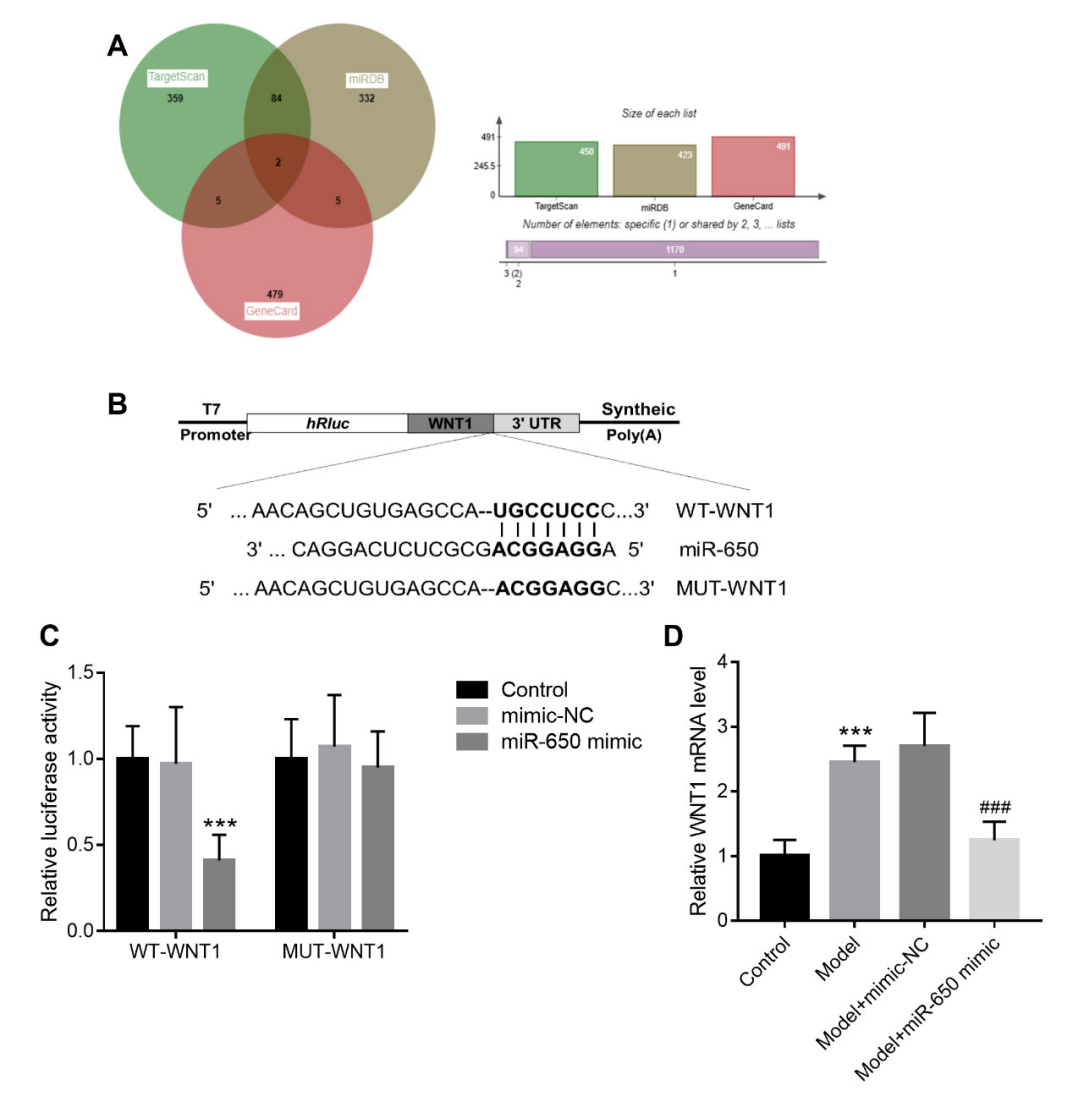
miR-650 directly targets WNT1 binding. Figure A: Overlapping target genes of miR-650 from the TargetScan and miRDB miRNA databases and from the GeneCards database. Figure B: Binding sequences between miR-650 and WNT1. Figure C: Luciferase activity of 293T cells transfected with miR-650 mimic or mimic-NC. Figure D: WNT1 mRNA levels in SW1353 cells. *** P < 0.001 vs. control group; ### < 0.001 vs. model group.

 The target genes of miR-650 were analyzed using the TargetScan and miRDB databases. A total of 450 target genes were identified from TargetScan, while 423 were identified from miRDB ( Figure 3 A). In addition, 491 OA-related targets were obtained from the GeneCards database ( Figure 3 A). Venn overlap analysis identified two overlapping target genes from the TargetScan, miRDB and GeneCards databases, namely IL1RN and WNT1 ( Figure 3 A). Based on the close association of WNT1 with OA, WNT1 was identified as a candidate target gene of miR-650 for further analysis. Figure 3 B shows the binding sequences between miR-650 and WNT1. According to the luciferase activity assay results, miR-650 overexpression weakened the luciferase activity of cells transfected with WT-MNT1, but no changes were detected in cells transfected with MUT-WNT1 ( Figure 3 C). Moreover, the downregulation of WNT1 mRNA levels was also tested in cells transfected with miR-650 mimic ( Figure 3 D). 

### WNT1 overexpression counteracted the role of miR-650 in SW1353 function

**Figure 4.**
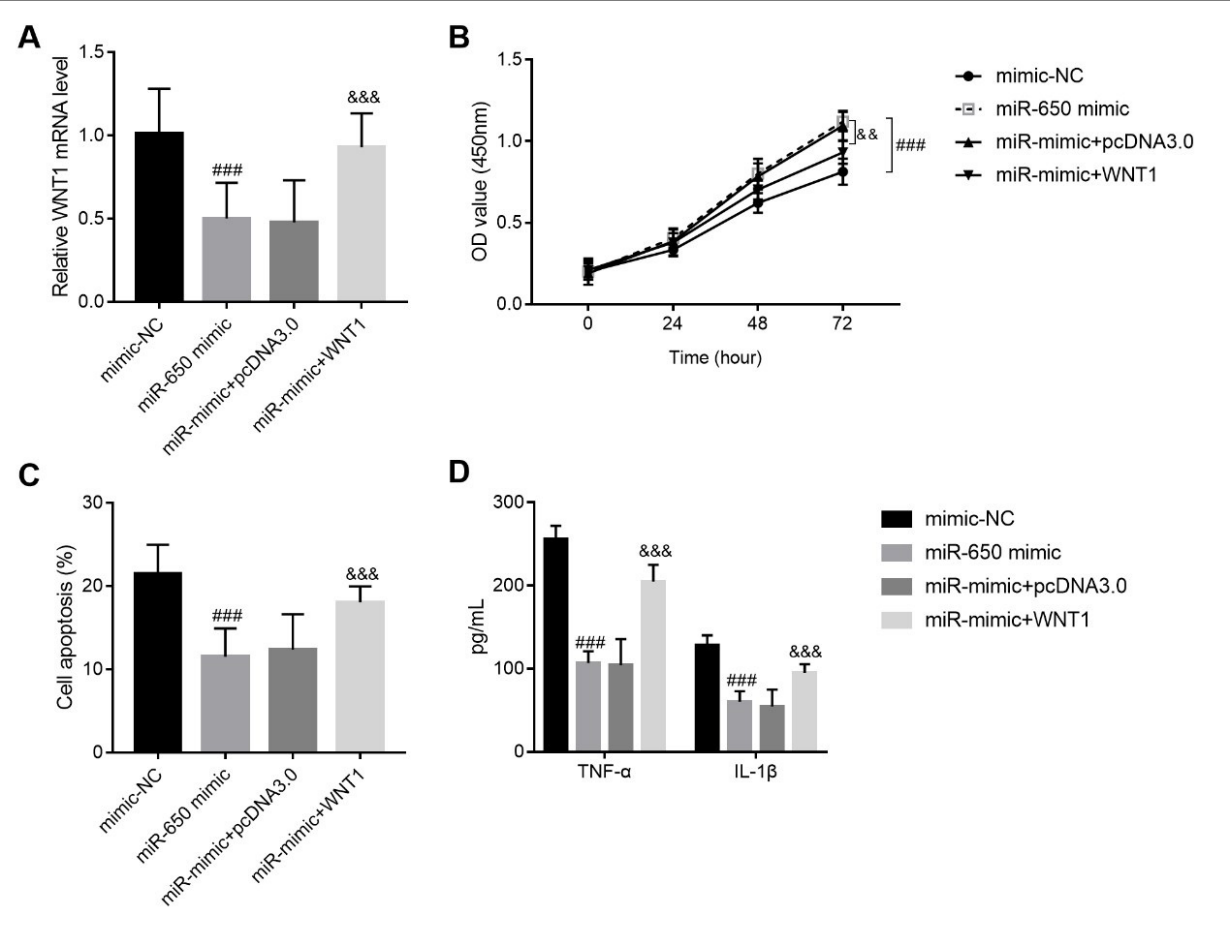
WNT1 overexpression counteracted the role of miR-650 in SW1353 function. Figure A: pcDNA 3.0-WNT1 transfection upregulates the mRNA level of WNT1. Figure B: Cell proliferation of OA cell models after transfection with miR-650 mimic or/and pcDNA-WNT1. Figure C: Cell apoptosis of OA cell models after transfection with miR-650 mimic or/and pcDNA-WNT1. Figure D: Concentration of TNF-α and IL-1β in OA cell models after transfection with miR-650 mimic or/and pcDNA-WNT1. ### P < 0.001 vs. mimic-NC group; &&& P < 0.001 vs. miR-650 mimic group.

 The involvement of WNT1 in miR-650 was also investigated in SW1353 cells, and its expression was mediated by pcDNA 3.0-WNT1 transfection. Figure 4 A shows its transfection efficiency, and an obvious increase of WNT1 mRNA levels was identified in cells after transfection of pcDNA 3.0-WNT1. In terms of cell function, overexpression of WNT1 resulted in the suppression of cell proliferation and promotion of cell apoptosis, which counteracted the effect of miR-650 on cells ( Figure 4 B-C). A similar effect was found in terms of inflammation, as an excessive release of both TNF-α and IL-1β was detected with the upregulation of WNT1 ( Figure 4 D). 

## DISCUSSION

 OA is the most common chronic degenerative joint disease, and its molecular mechanism is still not fully understood. ^15^ Recent studies have confirmed that miRNAs can be involved in chondrogenesis, cartilage degradation, and OA development by regulating cellular processes such as apoptosis, proliferation, and matrix remodeling. ^8^ For example, miR-17 has recently been reported to maintain cartilage homeostasis, which contributes to the prevention of OA. ^16^ In both OA cell and mice models, reduced miR-214-3p was found to be in good agreement with unbalanced extracellular matrix (ECM) metabolism in cartilage, and this mechanism is related to the activation of the NF-κB signaling pathway. ^17^ In the present study, miR-650 was specifically selected because of the prominent change in its expression level in inflamed synovial membrane after anterior cruciate ligament and/or meniscus injuries, based on the GE database. Moreover, miR-650 levels were analyzed clinically in cartilage tissues of patients with OA. As expected, suppressed miR-650 was detected in these cartilage tissues. Therefore, it was concluded that miR-650 may be a factor contributing to the development of OA. 

 Chondrocytes are important cellular structures for maintaining the structure and function of cartilage. ^18^ An increasing number of studies have shown that chondrocyte proliferation and apoptosis are involved in the onset of OA. ^19^ The death of chondrocytes disrupts the balance between extracellular matrix synthesis and degradation, further aggravating OA. ^20^ In this study, SW1353 cells were used to explore cell function, and IL-1β was applied to the cells to mimic OA conditions in vitro. Consistent with the results of previous studies, IL-1β treatment led to chondrocyte apoptosis and inhibition of cell proliferation. Moreover, the qRT-PCR results revealed the downregulation of miR-650 along with the prolongation of incubation time in SW1353 cells. To investigate the role of miR-650 in OA, miR-650 mimic was transfected into SW1353 to mediate its levels in vitro. As expected, miR-650 overexpression significantly promoted cell proliferation and suppressed apoptosis of chondrocytes, thereby counteracting the adverse effects of IL-1β. Furthermore, excessive release of inflammatory cytokines was detected in chondrocyte cell models. Inflammatory response is involved in the process of cartilage destruction in OA. ^21^ The in vitro experiments showed the anti-inflammatory role of miR-650 in chondrocytes, which is in agreement with the improved cell viability. It was concluded that the protective role of miR-650 against articular cartilage injury may be related to its anti-inflammatory effect. Consistently, the increase in miR-650 expression has been reported to suppress the release of inflammatory factors in the progression of rheumatoid arthritis ^13^ . In addition, it has been found to exert anti-inflammatory media in several other human diseases, such as ulcerative colitis. ^22^^,^^23^ The previous findings support our conclusion about OA. 

 Subsequently, the TargetScan and miRDB databases were used to investigate the downstream targets of miR-650, and 86 overlapping target genes were identified. In addition, 491 OA-related targets from the GeneCards database were identified by Venn overlap analysis, and two overlapping target genes were identified from the TargetScan, miRDB, and GeneCards databases. Among them, we focused on WNT family member 1 (WNT1), a key gene that plays a pivotal role in the regulation of OA. ^24^ In the progression of OA, the activation of WNT signaling can aggravate chondrocyte senescence. ^24^^,^^25^ In our cell experiments, the target association was confirmed by luciferase activity assay. Furthermore, the involvement of WNT1 in the role of miR-650 was investigated in SW1353 cells. It was found that WNT1 overexpression could partially abrogate the protective influence of miR-650 induction in response to articular cartilage injury in OA cell models. 

## CONCLUSION

In conclusion, our results showed that miR-650 can protect against articular cartilage injury in OA by targeting WNT1. This discovery provides a new pathway for exploring the pathogenesis of OA. However, the functions and mechanisms of the miR-650/WNT1 axis in OA need to be further verified in vivo.

## References

[1] Yi H., Zhang W., Cui S. Y., Fan J. B., Zhu X. H., Liu W. (2021). Identification and validation of key long non-coding RNAs in resveratrol protect against IL-1β-treated chondrocytes via integrated bioinformatic analysis. J Orthop Surg Res.

[2] Wu Z., Wang Y., Yan G., Wu C. (2023). Eugenol protects chondrocytes and articular cartilage by downregulating the JAK3/STAT4 signaling pathway. J Orthop Res.

[3] Abo-Zalam H. B., Abdelsalam R. M., Abdel-Rahman R. F., Abd-Ellah M. F., Khattab M. M. (2021). In Vivo Investigation of the Ameliorating Effect of Tempol against MIA-Induced Knee Osteoarthritis in Rats: Involvement of TGF-β1/SMAD3/NOX4 Cue. Molecules.

[4] Shi J., Cao F., Chang Y., Xin C., Jiang X., Xu J. (2021). Long non-coding RNA MCM3AP-AS1 protects chondrocytes ATDC5 and CHON-001 from IL-1beta-induced inflammation via regulating miR-138-5p/SIRT1. Bioengineered.

[5] Black A. L., Haskins J., Pozzi A., Clark A. L. (2023). Sexual dimorphism in reactive oxygen species production and a role for integrin α1β1 in estrogen receptor α and β expression in articular cartilage. J Orthop Surg Res.

[6] Zacharjasz J., Mleczko A. M., Bakowski P., Piontek T., Bakowska-Zywicka K. (2021). Small Noncoding RNAs in Knee Osteoarthritis: The Role of MicroRNAs and tRNA-Derived Fragments. Int J Mol Sci.

[7] Sondag G. R., Haqqi T. M. (2016). The Role of MicroRNAs and Their Targets in Osteoarthritis. Curr Rheumatol Rep.

[8] Huang P. Y., Wu J. G., Gu J., Zhang T. Q., Li L. F., Wang S. Q. (2021). Bioinformatics analysis of miRNA and mRNA expression profiles to reveal the key miRNAs and genes in osteoarthritis. J Orthop Surg Res.

[9] Ntoumou E., Tzetis M., Braoudaki M., Lambrou G., Poulou M., Malizos K. (2017). Serum microRNA array analysis identifies miR-140-3p, miR-33b-3p and miR-671-3p as potential osteoarthritis biomarkers involved in metabolic processes. Clin Epigenetics.

[10] Ye X., Lu Q., Yang A., Rao J., Xie W., He C. (2021). MiR-206 regulates the Th17/Treg ratio during osteoarthritis. Mol Med.

[11] Ren T., Wei P., Song Q., Ye Z., Wang Y., Huang L. (2020). MiR-140-3p Ameliorates the Progression of Osteoarthritis via Targeting CXCR4. Biol Pharm Bull.

[12] Wu W., Xuan Y., Ge Y., Mu S., Hu C., Fan R. (2021). Plasma miR-146a and miR-365 expression and inflammatory factors in patients with osteoarthritis. Malays J Pathol.

[13] Qu W., Jiang L., Hou G. (2021). Circ-AFF2/miR-650/CNP axis promotes proliferation, inflammatory response, migration, and invasion of rheumatoid arthritis synovial fibroblasts. J Orthop Surg Res.

[14] Xiao X., Yang X., Ren S., Meng C., Yang Z. (2022). Construction and analysis of a lncRNA-miRNA-mRNA competing endogenous RNA network from inflamed and normal synovial tissues after anterior cruciate ligament and/or meniscus injuries. Front Genet.

[15] Yu C. X., Sun S. (2018). An Emerging Role for Circular RNAs in Osteoarthritis. Yonsei Med J.

[16] Zhang Y., Li S., Jin P., Shang T., Sun R., Lu L. (2022). Dual functions of microRNA-17 in maintaining cartilage homeostasis and protection against osteoarthritis. Nat Commun.

[17] Cao Y., Tang S., Nie X., Zhou Z., Ruan G., Han W. (2021). Decreased miR-214-3p activates NF-κB pathway and aggravates osteoarthritis progression. EBioMedicine.

[18] Jenei-Lanzl Z., Meurer A., Zaucke F. (2019). Interleukin-1β signaling in osteoarthritis - chondrocytes in focus. Cell Signal.

[19] Li B., Guan G., Mei L., Jiao K., Li H. (2021). Pathological mechanism of chondrocytes and the surrounding environment during osteoarthritis of temporomandibular joint. J Cell Mol Med.

[20] Charlier E., Deroyer C., Ciregia F., Malaise O., Neuville S., Plener Z. (2019). Chondrocyte dedifferentiation and osteoarthritis (OA). Biochem Pharmacol.

[21] Vasconcelos D. P., Jabangwe C., Lamghari M., Alves C. J. (2022). The Neuroimmune Interplay in Joint Pain: The Role of Macrophages. Front Immunol.

[22] Li Y., Tang M., Zhang F. J., Huang Y., Zhang J., Li J. (2022). Screening of ulcerative colitis biomarkers and potential pathways based on weighted gene co-expression network, machine learning and ceRNA hypothesis. Hereditas.

[23] Xu X., Zhu X., Wang C., Li Y., Fan C., Kao X. (2019). microRNA-650 promotes inflammation induced apoptosis of intestinal epithelioid cells by targeting NLRP6. Biochem Biophys Res Commun.

[24] Li W., Xiong Y., Chen W., Wu L. (2020). Wnt/β-catenin signaling may induce senescence of chondrocytes in osteoarthritis. Exp Ther Med.

[25] Gu Y., Ren K., Wang L., Yao Q. (2019). Loss of Klotho contributes to cartilage damage by derepression of canonical Wnt/β-catenin signaling in osteoarthritis mice. Aging.

